# Tissue factor expression in breast cancer tissues: its correlation with prognosis and plasma concentration

**DOI:** 10.1054/bjoc.2000.1272

**Published:** 2000-06-15

**Authors:** T Ueno, M Toi, M Koike, S Nakamura, T Tominaga

**Affiliations:** Department of Surgery, Department of Pathology, Tokyo Metropolitan Komagome Hospital, 3–18–22, Honkomagome, Tokyo, Bunkyo-ku, 113–0021, Japan; Department of Cellular and Molecular Biology, Primate Research Institute, Kyoto University, Kanbayashi, Inuyama, 484–8506, Japan

**Keywords:** tissue factor, breast cancer, coagulation cascade, prognosis, metastasis

## Abstract

Tissue factor (TF), an initiator of the extrinsic coagulation cascade, is expressed in a wide range of cancer cells and plays important roles in cancer progression and metastasis. Recently, the intracellular function of TF has been revealed to be involved in cancer invasion, independent of the blood coagulation pathway. To evaluate the clinical significance of TF expression, we performed an enzyme-linked immunosorbent assay (ELISA) in the plasma of 67 breast cancer patients and immunohistochemistry in 213 breast cancer tissues. In the ELISA study, we showed an up-regulation of plasma TF concentration in breast cancer patients compared with normal controls. Immunohistochemistry demonstrated that TF was expressed in tumour cells and stromal cells and tumour TF expression closely correlated with stromal TF expression (*P* = 0.0005). The concentration of plasma TF was associated with tissue TF expression in both tumour and stroma. The multivariate analysis demonstrated that tumour TF expression was an independent prognostic indicator for overall survival (*P* = 0.0452). Our data show that plasma TF concentration reflects tissue TF expression and tumour TF expression can provide some predictive value for prognosis and distant metastasis, which indicates the importance of TF function in tumour progression. © 2000 Cancer Research Campaign


*British Journal of Cancer* (2000) **83**(2), 164–170
© 2000 Cancer Research Campaign
doi: 10.1054/ bjoc.2000.1272, available online at http://www.idealibrary.com on IDEAL®

### Tissue factor expression in breast cancer tissues: its correlation with prognosis and plasma concentration

**T Ueno[1], M Toi[1], M Koike[2], S Nakamura[3] and T Tominaga[1]**

[1]Department of Surgery, [2]Department of Pathology, Tokyo Metropolitan Komagome Hospital, 3–18–22, Honkomagome, Bunkyo-ku, Tokyo, 113–0021, Japan; [3]Department of Cellular and Molecular Biology, Primate Research Institute, Kyoto University, Kanbayashi, Inuyama, Aich, 484–8506, Japan

**Summary** Tissue factor (TF), an initiator of the extrinsic coagulation cascade, is expressed in a wide range of cancer cells and plays important roles in cancer progression and metastasis. Recently, the intracellular function of TF has been revealed to be involved in cancer invasion, independent of the blood coagulation pathway. To evaluate the clinical significance of TF expression, we performed an enzyme-linked immunosorbent assay (ELISA) in the plasma of 67 breast cancer patients and immunohistochemistry in 213 breast cancer tissues. In the ELISA study, we showed an up-regulation of plasma TF concentration in breast cancer patients compared with normal controls. Immunohistochemistry demonstrated that TF was expressed in tumour cells and stromal cells and tumour TF expression closely correlated with stromal TF expression ($P = 0.0005$). The concentration of plasma TF was associated with tissue TF expression in both tumour and stroma. The multivariate analysis demonstrated that tumour TF expression was an independent prognostic indicator for overall survival ($P = 0.0452$). Our data show that plasma TF concentration reflects tissue TF expression and tumour TF expression can provide some predictive value for prognosis and distant metastasis, which indicates the importance of TF function in tumour progression. © 2000 Cancer Research Campaign

**Keywords**: tissue factor; breast cancer; coagulation cascade; prognosis; metastasis

Tissue factor (TF) is a 47-kd glycoprotein expressed on the cell surface and a biological initiator of the extrinsic coagulation pathway as a receptor of factor VII/VIIa (FVII/VIIa) (Nemerson and Bach, 1982; Morrissey et al, 1987). Under physiological conditions, TF is expressed only in extravascular cells, including vascular adventitia, epidermis, mucosal epithelium, and alveolar macrophages. Some intravascular cells such as vascular endothelium and monocytes express TF when they are stimulated by several mediators, including bacterial lipopolysaccharides (LPS), thrombin, CD40 ligand (CD40L), and cytokines (Colucci et al, 1983; Bevilacqua et al, 1984; 1986; Conkling et al, 1988; Drake et al, 1989; Miller et al, 1998; Slupsky et al, 1998; Zhou et al, 1998). In cancer tissues, TF expression was reported to be observed in cancer cells as well as stromal cells by immunohistochemistry (Zacharski et al, 1983; Wojtukiewicz et al, 1989; Callander et al, 1992).

The coagulation cascade has long been investigated in the role of cancer progression and metastasis. Inhibition of thrombin by desulfatohirudin blocked experimental metastasis in mice and blocking of TF, factor Xa, or thrombin inhibited haematogenous metastasis in SCID mice (Esumi et al, 1991; Fischer et al, 1995). These observations emphasized the roles of the coagulation cascade in cancer metastasis.

Recently, many investigations demonstrated the important roles of TF function in cancer angiogenesis and metastasis, not only by its proteolytic activity via the coagulation cascade, but also by its intracellular signalling. It has been found that TF enhances vascular endothelial growth factor (VEGF) release (Zhang et al, 1994; Ollivier et al, 1998). In non-small cell lung carcinoma, the correlation was demonstrated between TF expression and angiogenic indicators exemplified by microvessel density (MVD) and VEGF expression, and co-localization of TF and VEGF was shown in both lung cancer and breast cancer cells (Koomägi and Volm, 1998; Shoji et al, 1998). TF expression in melanoma cells induced metastasis independent of the blood coagulation pathway (Bromberg et al, 1995), and, to the contrary, inhibition of TF function in human melanoma cells led to reduced haematogenous metastasis (Mueller et al, 1992). These findings suggest that TF is deeply involved in tumour progression both as an initiator of the coagulation pathway and as a mediator of signal transduction.

In the present study, we assessed plasma and tissue TF expression in breast cancer patients and showed its clinical significance, especially the prognostic value of TF.

*Received 9 June 1999*
*Revised 20 March 2000*
*Accepted 27 March 2000*

*Correspondence to:* M Toi

### PATIENTS AND METHODS

### Patients

Two hundred and thirteen primary breast cancer patients, 24 recurrent breast cancer patients, and 18 benign breast disease patients treated at Tokyo Metropolitan Komagome Hospital from 1983–1996 were enrolled in this study. All patients were female. All primary cancer patients had no distant metastases. Patients with liver and renal dysfunction were excluded from this study. Informed consent was obtained from all. The patients' age ranged from 29–85 years, and the average age was 52.3. Clinical stages were determined according to the criteria of the Japanese Breast Cancer Society which is based on the Union Internationale Contre le Cancer (UICC) criteria. All primary patients received either mastectomy or partial mastectomy with axillary lymph-node dissection. Adjuvant treatments were applied according to the following criteria which did not take account of the TF expression levels: polychemotherapy was given for 6 months to patients under the age of 55 with metastasized lymph nodes and hormonal therapy (tamoxifen) was given to oestrogen receptor (OR)-positive patients for more than 2 years. In the study, 173 patients received adjuvant therapy, including 53 chemotherapy, 38 hormonal therapy, and 82 chemo-endocrine therapy. For all patients, post-operative physical examinations were performed at least every 3 months. The median follow-up period of surviving patients was 53 months. Liver, lung, brain and distant lymph-node metastases were diagnosed using computed tomographic scan, and bone metastasis was diagnosed using X-ray and bone scintigraphy. In 24 recurrent cancer patients, major recurrent sites were found in liver ($n$ = 4), lung ($n$ = 1), bone ($n$ = 5), and lymph node ($n$ = 14). All recurrent patients were provided with the following treatments: nine with systemic chemotherapy, four with hormonal therapy, eight with chemo-endocrine therapy, and three with surgical resection. Eighteen benign breast disease patients included nine fibroadenoma, five mastopathy, and four intraductal papilloma.

### ELISA

Using plasma from 24 healthy female controls, 18 benign breast disease patients, 43 primary and 24 recurrent breast cancer patients who were treated from 1995–1996, circulating TF concentrations were examined. Venous blood samples were drawn into sterile vacuum tubes with 3.2% sodium citrate at the time of the diagnosis of primary breast cancer or recurrent cancer before treatments. They were centrifuged at 3000 rpm for 10 min and stored at –20°C until use. The TF level in the plasma was determined by enzyme-linked immunosorbent assay (ELISA) (Bobrow et al, 1989; Nakamura et al, 1993). This ELISA system adopted a sandwich method using two anti-TF monoclonal antibodies (mAbs) (HTF-K14, HTF-K180). Ninety six-well plates were coated with HTF-K14 and blocked with bovine serum albumin (BSA, Fraction V, Bayer, Germany). After incubation, HTF-K180 conjugated with horseradish peroxidase (HRP) was added. Then, to amplify the sensitivity, biotinyl-tyramide (Dupont)/$H_2O_2$ was added, followed by incubation with HRP-conjugated streptavidin (Amersham). After washing, 2,2′-azino-bis (3-athylbenz-thiazoline-6-sulfonic acid) (Boehringer Mannheim Biochemica)/$H_2O_2$ was added as a substrate. The reaction was stopped with 0.01% $NaN_3$ 0.1 mol $l^{-1}$ citric acid and absorbance at 420 nm was measured with the reference absorbance at 630 nm. Tissue factor was highly purified from human placenta with an anti-tissue factor antibody (HTF-K14) column and used as a standard for tissue factor ELISA. Detection limit of this ELISA was 10 pg $ml^{-1}$ (Nakamura et al, 1993).

### Immunohistochemistry

To assess TF expression, 3–5 μm sections of 213 paraffin-embedded breast cancer tissues, containing tissues from all patients whose plasma TF levels were measured, were applied to indirect anti-peroxidase immunohistochemical assay (Dako, CA, USA) using an anti-TF mAb (HTF-K108) (Imamura et al, 1993). After the inhibition of endogenous peroxidase activity, sections were stained with HTF-K108, followed by reaction with peroxidase-labelled anti-mouse immunoglobulin (Dako, CA, USA).

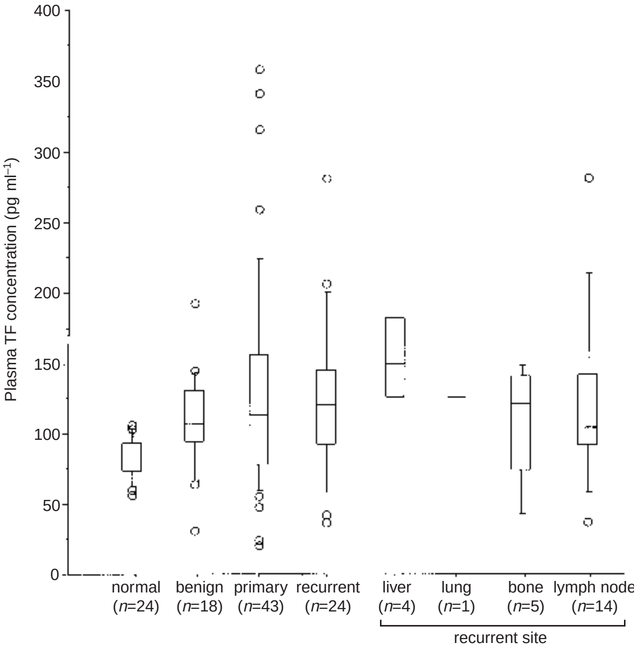

**Figure 1** Plasma TF concentration in normal controls, benign breast disease patients, and breast cancer patients. Primary and recurrent breast cancer patients had a significantly higher TF concentration than normal controls ($P$ = 0.0014 and 0.0114, respectively). There was no difference between normal controls and benign breast disease patients ($P$ = 0.12) and between primary and recurrent breast cancer patients ($P$ = 0.71). Among recurrent sites, no significant difference was observed in plasma TF concentration ($P$ = 0.68)

Peroxidase activity was visualized using 3,3′-diaminobenzidine (Nakalai Tesque, Kyoto, Japan) as substrate. After immunostaining, the sections were counterstained with haematoxylin (Muto Pure Chemicals Co, Ltd, Tokyo, Japan).

TF-positive cancer cells (tumour TF) and stromal cells (stromal TF) were separately counted in the most accumulated five areas in a × 200 microscopic field. In each of cancer and stroma, the average of the counts was regarded as TF-positive cell density, which was classified into three groups: – = < 10%, + = 10–50%, ++ = > 50%. The immunohistochemical and pathological assessment was carried out by two pathologists who had no information of clinical conditions and plasma TF levels.

### Hormone receptor assay

Oestrogen receptor (OR) and progesterone receptor (PgR) were measured by dextran-coated charcoal (DCC) method using $^3$H-17 oestradiol or by EIA method. The cut-off values were determined as 5 fmol $mg^{-1}$ protein for both hormone receptors.

### Statistical analysis

Plasma TF concentration was compared using analysis of variance (ANOVA) and Fisher's protected least-significant difference (PLSD). The chi-square test was carried out for qualitative analysis. The Spearman's correlation coefficient by rank was performed to evaluate the correlation between tissue TF expression and plasma TF concentration. The survival curves were drawn by Kaplan–Meier method, and the difference in prognosis between two groups was analysed by the log-rank test. The

© 2000 Cancer Research Campaign

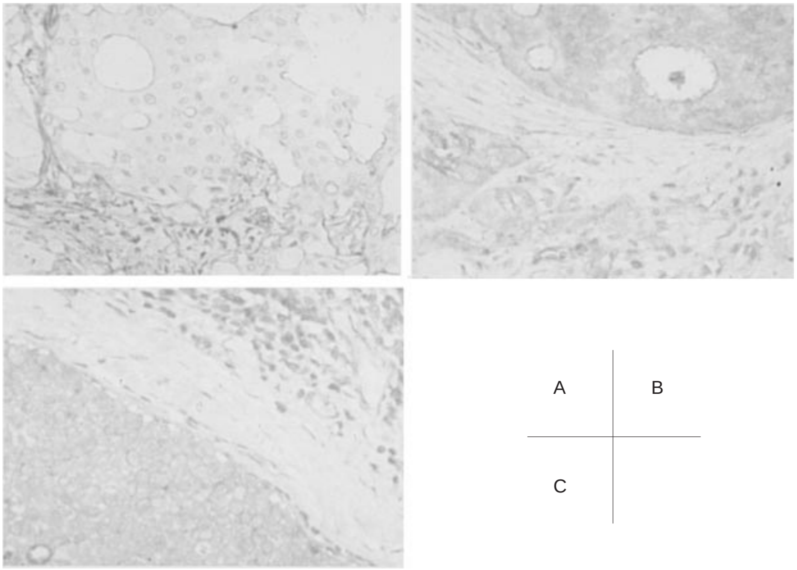

**Figure 2** Immunohistochemical studies of TF expression in breast cancer tissues. TF was expressed in both tumour cells and stromal cells, including fibroblastic cells, monocytic cells, and endothelial cells. (**A**) tumour TF –, stroma TF ++; (**B**) tumour TF +, stroma TF +; (**C**) tumour TF ++, stroma TF ++

relative risk for each indicator was evaluated by the multivariate Cox proportional hazards model. $P < 0.05$ was considered to indicate statistical significance.

### RESULTS

### ELISA

The median levels of plasma TF concentration in normal female controls, benign breast disease, and primary and recurrent breast cancer patients were 83.85 (25th–75th percentile: 72.40–92.55), 106.1 (94.03–131.3), 111.6 (77.33–155.4), and 120.1 (92.02–145.0) pg $ml^{-1}$ respectively (Figure 1). Statistical analysis demonstrated that primary and recurrent breast cancer patients showed significantly higher plasma TF concentration than normal controls ($P$ = 0.0014 and 0.0114, respectively), whereas there was no difference between normal controls and benign breast disease patients ($P$ = 0.12), and between primary and recurrent breast cancer patients ($P$ = 0.71). In primary cancer patients, plasma TF concentration was not significantly associated with any clinical parameters including clinical stage, tumour size, lymph-node metastasis, hormone receptor status and pathological findings (data not shown). In the case of recurrent cancer patients, although patients with liver metastasis tended to have high plasma TF concentration, there was no statistical difference among patients with different recurrent sites ($P$ = 0.68, Figure 1).

### Immunohistochemical study

TF expression was observed in tumour cells, fibroblastic cells, monocytic cells, and vascular endothelial cells (Figure 2). Among 213 primary breast cancer tissues, tumour TF expression was detected in 193 (90.6%) tissues including 117 + and 76 ++, and stromal TF expression was detected in almost all tissues (210 tissues, 98.6%) (Figure 2, Table 1). Tumour TF expression closely correlated with stromal TF expression ($P$ = 0.0005, Table 1). Table 1 shows that both tumour and stromal TF expression were not associated with any clinical parameters, such as clinical stage, hormone-receptor status, lymph-node metastasis, and adjuvant treatments. We assessed the relationship between plasma TF concentration and TF expression in cancer tissues. Plasma TF concentration was significantly associated with staining intensity of TF in cancer tissues. Figure 3 shows that there was a close correlation between plasma TF levels and TF expression, not only in tumour cells but also stromal cells ($P$ = 0.0069 and 0.0003, respectively). Survival analysis revealed that tumour TF-negative patients ($n$ = 20) had a significantly better prognosis than tumour TF-positive patients ($n$ = 193) for overall survival ($P$ = 0.041), although no significant difference was shown for disease-free survival ($P$ = 0.266) (Figure 4). Moreover, tumour TF expression was proved to be an independent prognostic indicator for overall survival by the multivariate analysis (hazard ratio = 4.275, $P$ = 0.0452, Table 2). During the follow-up period, 85 patients underwent recurrence, and recurrent sites are shown in Table 3. Among tumour TF-negative patients (mean follow-up period = 84 months, range = 1–175 months), no metastases in liver, lung, and brain were observed, whereas 36.7% of tumour TF-positive patients (mean follow-up period = 58 months, range = 7–173 months) underwent metastases in those sites.

### DISCUSSION

Plasma TF concentration has been reported to be increased in various disease such as disseminated intravascular coagulation (DIC), ischaemic heart disease, antiphospholipid syndrome, and cancer (Koyama et al, 1994; Takahashi et al, 1994; Wada et al, 1994; Kakkar et al, 1995; Suefuji et al, 1997; Cuadrando et al, 1998; Falciani et al, 1998; Misumi et al, 1998). We showed that plasma TF concentration was up-regulated in primary and recurrent breast cancer patients. In glioblastoma cells, shedding of TF was shown, suggesting that increased concentration of TF in cancer patients may result from TF emission from tumour cells as well as stromal cells (Bastida et al, 1994). We demonstrated that there was a significant correlation between plasma TF

 © 2000 Cancer Research Campaign

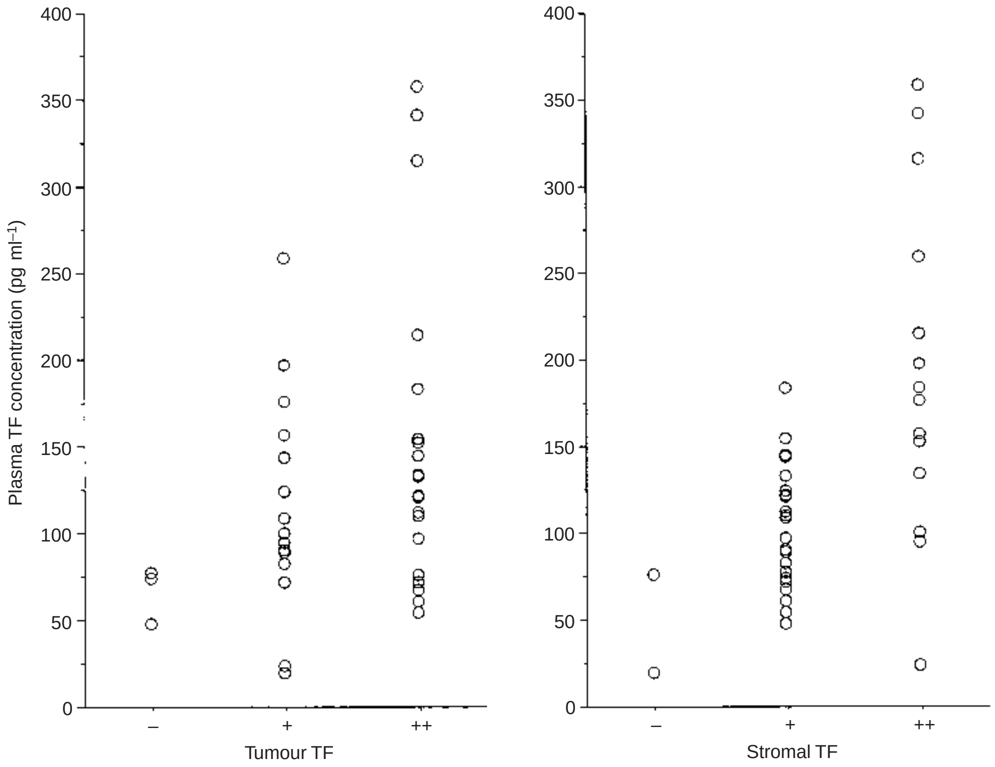

**Figure 3** Correlation between TF expression in cancer tissues and plasma TF concentration. Both tumour and stromal TF expression were significantly associated with plasma TF concentration (*P* = 0.0069 and 0.0003, respectively).

**Table 1** Background analysis

| Clinical parameters | Tumour TF – (*n* = 20) | Tumour TF + (*n* = 117) | Tumour TF ++ (*n* = 76) | *P* | Stromal TF – (*n* = 3) | Stromal TF + (*n* = 101) | Stromal TF ++ (*n* = 109) | *P* |
|---|---|---|---|---|---|---|---|---|
| Clinical stage | | | | | | | | |
| I | 4 | 25 | 25 | | 2 | 25 | 27 | |
| II | 11 | 57 | 37 | NS | 1 | 50 | 54 | NS |
| III | 4 | 27 | 9 | | 0 | 19 | 21 | |
| IV | 1 | 5 | 3 | | 0 | 5 | 4 | |
| Menopausal status | | | | | | | | |
| pre- | 9 | 43 | 38 | | 1 | 41 | 48 | |
| post- | 11 | 72 | 37 | NS | 2 | 58 | 60 | NS |
| Oestrogen receptor (OR) | | | | | | | | |
| – | 4 | 38 | 24 | | 0 | 26 | 40 | |
| + | 10 | 56 | 25 | NS | 1 | 43 | 47 | NS |
| unknown | 6 | 23 | 27 | | 2 | 32 | 22 | |
| Progesterone receptor (PgR) | | | | | | | | |
| – | 5 | 40 | 28 | | 1 | 31 | 41 | |
| + | 9 | 51 | 18 | NS | 0 | 36 | 42 | NS |
| unknown | 6 | 24 | 30 | | 2 | 33 | 25 | |
| Pathological parameters | | | | | | | | |
| Venous invasion (v) | | | | | | | | |
| – | 0 | 12 | 9 | | 1 | 8 | 12 | |
| 1+ | 16 | 71 | 42 | NS | 0 | 57 | 72 | NS |
| 2+ | 0 | 16 | 1 | | 0 | 6 | 11 | |
| 3+ | 0 | 1 | 0 | | 0 | 1 | 0 | |
| Nodal metastasis (*n*) | | | | | | | | |
| – | 7 | 34 | 24 | | 1 | 25 | 39 | |
| + | 9 | 66 | 28 | NS | 0 | 47 | 56 | NS |
| Adjuvant treatments | | | | | | | | |
| hormonal therapy | 8 | 15 | 15 | | 1 | 21 | 16 | |
| chemotherapy | 5 | 27 | 21 | NS | 0 | 29 | 24 | NS |
| chemo-endocrine | 5 | 53 | 24 | | 1 | 36 | 45 | |
| none | 2 | 22 | 16 | | 1 | 15 | 24 | |
| Stromal TF | | | | | | | | |
| – | 0 | 2 | 1 | | | | | |
| + | 17 | 60 | 24 | *P* = 0.0005 | | | | |
| ++ | 3 | 55 | 51 | | | | | |

NS, not significant..

*© 2000 Cancer Research Campaign* 

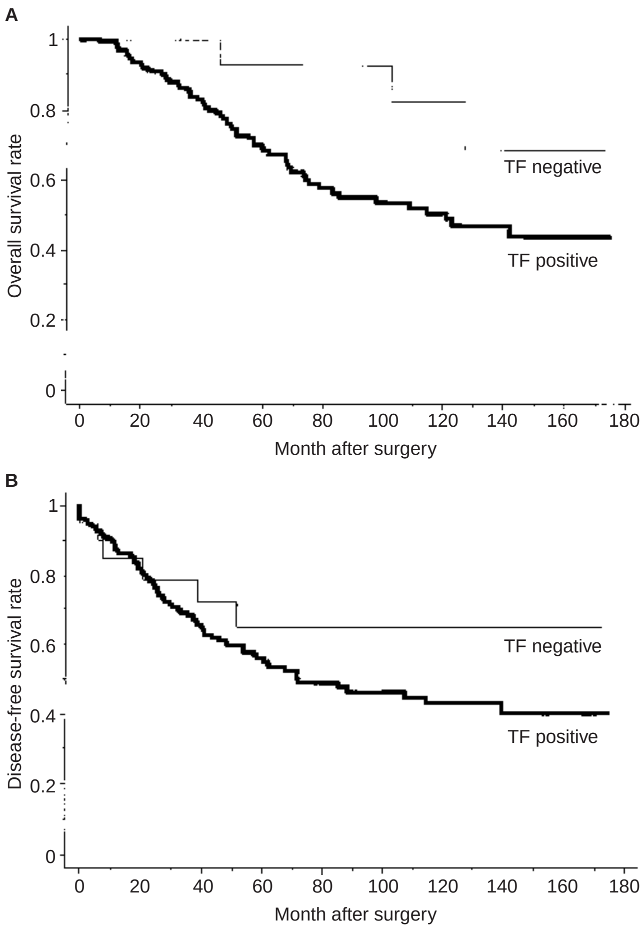

**Figure 4** (**A**) Overall survival curve in 213 primary breast cancer patients. Tumour TF-positive patients had a significantly worse prognosis than tumour TF-negative patients ($P = 0.041$). (**B**) Disease-free survival curve. There was no significant difference in prognosis between tumour TF-positive and -negative patients ($P = 0.266$).

**Table 2** Multivariate analysis for overall survival

| | Hazard ratio | 95% CI | *P* value |
|---|---|---|---|
| Tumour TF: +,++ vs – | 4.275 | 1.031–17.721 | 0.0452 |
| Oestrogen receptor: – vs + | 2.132 | 1.250– 3.650 | 0.0055 |
| Lymph node metastasis: + vs – | 3.086 | 1.629– 5.848 | 0.0005 |
| Tumour size: ≥ 2.0 cm vs < 2.0 cm | 1.062 | 0.367– 3.070 | 0.9116 |

**Table 3** Recurrent sites observed during the follow-up period

| | Tumour TF | | | Stromal TF | | |
|---|---|---|---|---|---|---|
| | – | + | ++ | – | + | ++ |
| Liver | 0 | 16 | 6 | 0 | 10 | 12 |
| Lung | 0 | 10 | 3 | 0 | 3 | 10 |
| Brain | 0 | 1 | 2 | 0 | 0 | 3 |
| Bone | 3 | 15 | 6 | 0 | 12 | 12 |
| Lymph node | 3 | 16 | 8 | 0 | 10 | 17 |

concentration and tissue TF expression in both tumour and stroma, supporting the view that TF expression in plasma derives from both tumour cells and stromal cells, including macrophages and endothelium, in cancer patients. Previously we showed that plasma TF in female controls was lower than that in male controls (Nakamura et al, 1993) and, in this study, we used female controls because all patients were female.

In the immunohistochemical study, we confirmed that TF was widely expressed in both tumour cells and stromal cells, and that tumour TF expression was a predictor of poor prognosis in breast cancer. It was reported that TF expression in tumour cells correlated with grade of malignancy in glioma and pancreatic cancer (Kakkar et al, 1995; Hamada et al, 1996). In addition, numerous studies have clarified the role of TF in metastasis and angiogenesis. In colorectal cancer, TF expression in metastatic liver tumours increased compared with primary tumours (Shigemori et al, 1998). Our data showed that tumour TF-positive and -negative patients differed in the distribution of recurrent sites. In particular, none of the tumour TF-negative patients had liver metastasis, although this result is preliminary. Liver metastasis is well-known to provide a poor prognosis in breast cancer patients (Yamamoto et al, 1998). Taken together, although no difference in prognosis was observed for disease-free survival, the difference in metastatic sites between tumour TF-positive and -negative patients might result in the significant difference in prognosis for overall survival. Since the number of tumour TF-negative patients was small, additional studies are needed to confirm our results. However, our data imply the importance of TF expression in breast cancer progression.

Several studies reported that TF was expressed in breast cancer cells, but the expression varied in degree from 37.5–100% among investigators (Callander et al, 1992; Contrino et al, 1996; Vrana et al, 1996). Our study showed that tumour TF-expression was observed in more than 90% of the breast cancer tissues. Although this discrepancy may result from differences in the sensitivity of antibodies, the preparation method of tissues, the condition of immunohistochemistry, and the criteria of TF-expression levels, our data coincides with recent studies showing that tumour TF-expression was observed in almost all breast cancer tissues (Contrino et al, 1996; Vrana et al, 1996).

Recently, several new findings on the role of tissue factor came out. The binding of FVIIa to TF induces intracellular calcium ion oscillation, tyrosine phosphorylation, up-regulation of poly-A polymerase mRNA and activation of the MAP kinase pathway, indicating that TF functions not only as a coagulation factor but also as a mediator of intracellular signaling (Rottingen et al, 1995; Masuda et al, 1996; Pendurthi et al, 1997; Poulsen et al, 1998). TF was reported to be associated with the γ chain homodimer of the IgE receptor type I in monocytes (Masuda et al, 1996). In addition, TF-FVIIa pathway was demonstrated to enhance urokinase receptor (uPAR) expression in cancer cells, leading to tumour invasion (Taniguchi et al, 1998). Two-hybrid screening revealed interaction of TF cytoplasmic domain with actin-binding protein 280 (ABP-280), which mediates cell adhesion and migration (Ott et al, 1998). These observations suggest that TF might modify the phenotype of cells into an invasive one by activation of certain signal transduction pathways. In fact, Chinese hamster ovary (CHO) cells transfected with TF were reported to acquire metastatic potential in SCID mice (Mueller and Ruf, 1998). This phenotypic change may contribute to a poor prognosis in TF-positive patients.

 *© 2000 Cancer Research Campaign*

In conclusion, this study demonstrated the association between the concentration of plasma TF and TF-expression in cancer tissues, and the significance of tumour TF-expression as an independent prognostic indicator for overall survival. It may contribute to a poor prognosis that TF promotes cancer invasion and metastasis both through activation of the blood coagulation cascade and through activation of certain intracellular signaling in TF-expressing cells. A clear identification of the extra- and intracellular function of TF will add greatly to the understanding of the mechanism of cancer invasion and metastasis.

### ACKNOWLEDGEMENTS

We thank Miss Mariko Muta and Miss Miwa Otsuka for their technical and statistical assistance and Mr George Chang for reviewing the manuscript. This work was supported in part by grant-in-aid for scientific research (c) (09671108 to SN) from the Ministry of Education, Science, Sports and Culture, Japan.

### REFERENCES

Bach R, Gentry R and Nemerson Y (1986) Factor VII binding to tissue factor in reconstituted phospholipid vesicles: induction of cooperativity by phosphatidylserine. *Biochemistry* **25**: 4007–4020

Bastida E, Ordinas A, Escolar G and Jamieson GA (1994) Tissue factor in microvesicles shed from U87MG human glioblastoma cells induces coagulation, platelet aggregation, and thrombogenesis. *Blood* **64**: 177–181

Bevilacqua MP, Pober JS, Majeau GR, Cotran RS and Glimbrone MA Jr (1984) Interleukin 1 induces biosynthesis and cell surface expression of procoagulant activity in human vascular endothelial cells. *J Exp Med* **160**: 618–623

Bevilacqua MP, Pober JS, Manjeau GR, Fiers W, Cotran RS and Gimbrone MA (1986) Recombinant tumour necrosis factor induces procoagulant activity in cultured human vascular endothelium: characterization and comparison with the actions of interleukin 1. *Proc Natl Acad Sci USA* **83**: 4533–4537

Bobrow MN, Harris TD and Shaughnessy KJ (1989) Catalyzed reporter deposition, a novel method of signal amplification; application to immunoassays. *J Immunol Methods* **125**: 279–285

Bromberg ME, Konigsberg WH, Madison JF, Pawashe A and Garen A (1995) Tissue factor promotes melanoma metastasis by a pathway independent of blood coagulation. *Proc Natl Acad Sci USA* **92**: 8205–8208

Brox JH, Osterud B, Bjorklid E and Fenton II JW (1984) Production and availability of thromboplastin in endothelial cells: the effects of thrombin, endotoxin and platelets. *Br J Haematol* **57**: 239–246

Callander NS, Varki N and Rao LVM (1992) Immunohistochemical identification of tissue factor in solid tumours. *Cancer* **70**: 1194–1201

Colucci M, Balconi G, Lorenzet R, Pietra A, Locati D, Donati MB and Semerara N (1983) Cultured human endothelial cells generate tissue factor in response to endotoxin. *J Clin Invest* **71**: 1893–1896

Conkling PR, Greenberg CS and Weinberg JB (1988) Tumour necrosis factor induces tissue factor-like activity in human leukemia cell line U939 and peripheral blood monocytes. *Blood* **72**: 128–133

Contrino J, Hair G, Kreutzer DL and Rickles FR (1996) In situ detection of tissue factor in vascular endothelial cells: correlation with the malignant phenotype of human breast disease. *Nat Med* **2**: 209–215

Cuadrando MJ, Dobado-Berrios PM, Lopez-Pedrera C, Gomez-Zumaquero JM, Tinahones F, Jurado A, Camps T, Torres A and Velasco F (1998) Variability of soluble tissue factor in primary antiphospholipid syndrome. *Thromb Haemost* **80**: 712–713

Drake TA, Morrissey JH and Edgington TS (1989) Selective cellular expression of tissue factor in human tissues. Implication for disorders of hemostasis and thrombosis. *Am J Pathol* **134**: 1087–1097

Esumi N, Fan D and Fidler IJ (1991) Inhibition of murine melanoma experimental metastasis by recombinant desulfatohirudin, a highly specific thrombin inhibitor. *Cancer Res* **51**: 4549–4556

Fadok V, Voelker D, Campbell P, Cohen J, Bratton D and Henson P (1992) Exposure of phosphatidylserine on the surface of apoptotic lymphocytes triggers specific recognition and removal by macrophages. *J Immunol* **148**: 2207–2216

Falciani M, Gori AM, Fedi S, Chiarugi L, Simonetti I, Dabizzi RP, Prisco D, Pepe G, Abbate R, Gensini GF and Neri Serneri GG (1998) Elevated tissue factor and tissue factor pathway inhibitor circulating levels in ischemic heart disease patients. *Thromb Haemost* **79**: 495–499

Fischer EG, Ruf W and Mueller BM (1995) Tissue factor-induced thrombin generation activates the signaling thrombin receptor on malignant melanoma cells. *Cancer Res* **55**: 1629–1632

Greeno EW, Bach R and Moldow CF (1996) Apoptosis is associated with increased cell surface tissue factor procoagulant activity. *Lab Invest* **75**: 281–289

Hamada K, Kuratsu J, Saitoh Y, Takeshima H, Nishi T and Ushio Y (1996) Expression of tissue factor correlates with grade of malignancy in human glioma. *Cancer* **77**: 1877–1883

Imamura T, Iyama K, Takeya M, Kambara T and Nakamura S (1993) Role of macrophage tissue factor in the development of the delayed hypersensitivity reaction in monkey skin. *Cell Immunol* **152**: 614–622

Kakkar AK, DeRuvo N, Chinswangwatanakul V, Tebbutt S and Williamson RC (1995) Extrinsic-pathway activation in cancer with high factor VIIa and tissue factor. *Lancet* **346**: 1004–1005

Kakkar AK, Lemoine NR, Scully MF, Tebbutt S and Williamson RCN (1995) Tissue factor expression correlates with histological grade in human pancreatic cancer. *Br J Surg* **82**: 1101–1104

Koomägi R and Volm M (1998) Tissue factor expression in human non-small cell lung carcinoma measured by immunohistochemistry: correlation between tissue factor and angiogenesis. *Int J Cancer* **79**: 19–22

Koyama T, Nishida K, Ohdama S, Sawada M, Murakami N, Hirosawa S, Kuriyama R, Matsuzawa K, Hasegawa R and Aoki N (1994) Determination of plasma tissue factor antigen and its clinical significance. *Br J Haematol* **87**: 343–347

Masuda M, Nakamura S, Murakami T, Komiyama Y and Takahashi H (1996) Association of tissue factor with a γ-chain homodimer of the IgE receptor type I in cultured human monocytes. *Eur J Immunol* **26**: 2529–2532

Miller DL, Yaron R and Yellin MJ (1998) CD40L-CD40 interactions regulate endothelial cell surface tissue factor and thrombomodulin expression. *J Leukoc Biol* **63**: 373–379

Misumi K, Ogawa H, Yasue H, Soejima H, Suefuji H, Nishiyama K, Takazoe K, Kugiyama K, Tsuji I, Kumeda K and Nakamura S (1998) Comparison of plasma tissue factor levels in unstable and stable angina pectoris. *Am J Cardiol* **81**: 22–26

Morrissey JH, Fakhrai H and Edgington TS (1987) Molecular cloning of the cDNA for tissue factor, the cellular receptor for the initiation of the coagulation protease cascade. *Cell* **50**: 129–135

Mueller BM, Reisfeld RA, Edgington TS and Ruf W (1992) Expression of tissue factor by melanoma cells promotes efficient hematogenous metastasis. *Proc Natl Acad Sci USA* **89**: 11832–11836

Mueller BM and Ruf W (1998) Requirement for binding of catalytically active factor VIIa in tissue factor-dependent experimental metastasis. *J Clin Invest* **101**: 1372–1378

Nakamura S, Kamikubo Y, Okajima K, Asakura H and Nakamura K (1993) Plasma tissue factor: its sensitive assay and characterization. *Thromb Haemost* **69**: 744

Nemerson Y and Bach R (1982) Tissue factor revisited. *Progress in Hemostasis and Thrombosis* **6**: 237–261

Ollivier V, Bentolila S, Chabbat J, Hakim J and de Prost D (1998) Tissue factor-dependent vascular endothelial growth factor production by human fibroblasts in response to activated factor VII. *Blood* **91**: 2698–2703

Ott I, Fischer EG, Miyagi Y, Mueller BM and Ruf W (1998) A role for tissue factor in cell adhesion and migration mediated by interaction with actin-binding protein 280. *J Cell Biol* **140**: 1241–1253

Pendurthi UR, Alock D and Rao LVM (1997) Binding of factor VIIa to tissue factor induces alterations in gene expression in human fibroblast cells: up-regulation of poly (A) polymerase. *Proc Natl Acad Sci USA* **94**: 12598–12603

Poulsen LK, Jacobsen N, Sorensen BB, Bergenhem NCH, Kelly JD, Foster DC, Thastrup O, Ezban M and Petersen LC (1998) Signal transduction via the mitogen-activated protein kinase pathway induced by binding of coagulation factor VIIa to tissue factor. *J Biol Chem* **273**: 6228–6232

Rottingen J-A, Enden T, Camerer E, Iversen J-G and Prydz H (1995) Binding of human factor VIIa to tissue factor induces cytosolic Ca2+ signals in J82 cells, transfected COS-1 cells, Madin-Darby canine kidney cells and in human endothelial cells induced to synthesize tissue factor. *J Biol Chem* **270**: 4650–4660

Shigemori C, Wada H, Matsumoto K, Shiku H, Nakamura S and Suzuki H (1998) Tissue factor expression and metastatic potential of colorectal cancer. *Thromb Haemost* **80**: 894–898

Shoji M, Hancock WW, Abe K, Micko C, Casper KA, Baine RM, Wilcox JN, Danave I, Dillehay DL, Matthews E, Contrino J, Morrissey JH, Gordon S, Edgington TS, Kudryk B, Kreutzer DL and Rickles FR (1998) Activation of

© 2000 Cancer Research Campaign coagulation and angiogenesis in cancer. Immunohistochemical localization in situ of clotting proteins and vascular endothelial growth factor in human cancer. *Am J Pathol* **152**: 399–411

Slupsky JR, Kalbas M, Willuweit A, Henn V, Kroczek RA and Muller-Berghaus G (1998) Activated platelets induce tissue factor expression on human umbilical vein endothelial cells by ligation of CD40. *Thromb Haemost* **80**: 1008–1014

Suefuji H, Ogawa H, Yasue H, Kaikita K, Soejima H, Motoyama T, Mizuno Y, Oshima S, Saito T, Tsuji I, Kumeda K, Kamikubo Y and Nakamura S (1997) Increased plasma tissue factor levels in acute myocardial infarction. *Am Heart J* **134**: 253–259

Takahashi H, Satoh N, Wada K, Takakuwa E, Seki Y and Shibata A (1994) Tissue factor in plasma of patients with disseminated intravascular coagulation. *Am J Hematol* **46**: 333–337

Taniguchi T, Kakkar AK, Tuddenham EGD, Williamson RCN and Lemoine NR (1998) Enhanced expression of urokinase receptor induced through the tissue factor–factor VIIa pathway in human pancreatic cancer. *Cancer Res* **58**: 4461–4467

Vrana JA, Stang MT, Grande JP and Getz MJ (1996) Expression of tissue factor in tumour stroma correlates with progression to invasive human breast cancer: paracrine regulation by carcinoma cell-derived members of the transforming growth factor β family. *Cancer Res* **56**: 5063–5070

Wada H, Nakase T, Nakaya R, Minamikawa K, Wakita Y, Kaneko T, Ohiwa M, Deguchi K and Shirakawa S (1994) Elevated plasma tissue factor antigen level in patients with disseminated intravascular coagulation. *Am J Hematol* **45**: 232–236

Wojtukiewicz MZ, Zacharski LR, Memoli VA, Kisiel W, Kudryk BJ, Rousseau SM and Stump DC (1989) Indirect activation of blood coagulation in colon cancer. *Thromb Haemost* **62**: 1062–1066

Yamamoto N, Watanabe T, Katsumata N, Omuro Y, Ando M, Fukuda H, Tokue Y, Narabayashi M, Adachi I and Takashima S (1998) Construction and validation of a practical prognostic index for patients with metastatic breast cancer. *J Clin Oncol* **16**: 2401–2408

Zacharski LR, Schned AR and Sorenson GD (1983) Occurrence of fibrin and tissue factor antigen in human small cell carcinoma of the lung. *Cancer Res* **43**: 3963–3968

Zhang Y, Deng Y, Thomas L, Muller M, Ziegler R, Waldherr R, Stern DM and Nawroth PP (1994) Tissue factor controls the balance of angiogenic and antiangiogenic properties of tumour cells in mice. *J Clin Invest* **94**: 1320–1327

Zhou L, Stordeur P, de Lavareille A, Thielemans K, Capel P, Goldman M and Pradier O (1998) CD40 engagement on endothelial cells promotes tissue factor-dependent procoagulant activity. *Thromb Haemost* **79**: 1025–1028

© 2000 Cancer Research Campaign

## References

[PDF_00485] Bach R., Gentry R., Nemerson Y. (1986). Factor VII binding to tissue factor in reconstituted phospholipid vesicles: induction of cooperativity by phosphatidylserine.. Biochemistry.

[PDF_00488] Bastida E., Ordinas A., Escolar G., Jamieson G. A. (1984). Tissue factor in microvesicles shed from U87MG human glioblastoma cells induces coagulation, platelet aggregation, and thrombogenesis.. Blood.

[PDF_00491] Bevilacqua M. P., Pober J. S., Majeau G. R., Cotran R. S., Gimbrone M. A. (1984). Interleukin 1 (IL-1) induces biosynthesis and cell surface expression of procoagulant activity in human vascular endothelial cells.. J Exp Med.

[PDF_00494] Bevilacqua M. P., Pober J. S., Majeau G. R., Fiers W., Cotran R. S., Gimbrone M. A. (1986). Recombinant tumor necrosis factor induces procoagulant activity in cultured human vascular endothelium: characterization and comparison with the actions of interleukin 1.. Proc Natl Acad Sci U S A.

[PDF_00498] Bobrow M. N., Harris T. D., Shaughnessy K. J., Litt G. J. (1989). Catalyzed reporter deposition, a novel method of signal amplification. Application to immunoassays.. J Immunol Methods.

[PDF_00501] Bromberg M. E., Konigsberg W. H., Madison J. F., Pawashe A., Garen A. (1995). Tissue factor promotes melanoma metastasis by a pathway independent of blood coagulation.. Proc Natl Acad Sci U S A.

[PDF_00504] Brox J. H., Osterud B., Bjørklid E., Fenton J. W. (1984). Production and availability of thromboplastin in endothelial cells: the effects of thrombin, endotoxin and platelets.. Br J Haematol.

[PDF_00507] Callander N. S., Varki N., Rao L. V. (1992). Immunohistochemical identification of tissue factor in solid tumors.. Cancer.

[PDF_00509] Colucci M., Balconi G., Lorenzet R., Pietra A., Locati D., Donati M. B., Semeraro N. (1983). Cultured human endothelial cells generate tissue factor in response to endotoxin.. J Clin Invest.

[PDF_00512] Conkling P. R., Greenberg C. S., Weinberg J. B. (1988). Tumor necrosis factor induces tissue factor-like activity in human leukemia cell line U937 and peripheral blood monocytes.. Blood.

[PDF_00515] Contrino J., Hair G., Kreutzer D. L., Rickles F. R. (1996). In situ detection of tissue factor in vascular endothelial cells: correlation with the malignant phenotype of human breast disease.. Nat Med.

[PDF_00518] Cuadrado M. J., Dobado-Berrios P. M., López-Pedrera C., Gómez-Zumaquero J. M., Tinahones F., Jurado A., Camps T., Torres A., Velasco F. (1998). Variability of soluble tissue factor in primary antiphospholipid syndrome.. Thromb Haemost.

[PDF_00522] Drake T. A., Morrissey J. H., Edgington T. S. (1989). Selective cellular expression of tissue factor in human tissues. Implications for disorders of hemostasis and thrombosis.. Am J Pathol.

[PDF_00525] Esumi N., Fan D., Fidler I. J. (1991). Inhibition of murine melanoma experimental metastasis by recombinant desulfatohirudin, a highly specific thrombin inhibitor.. Cancer Res.

[PDF_00528] Fadok V. A., Voelker D. R., Campbell P. A., Cohen J. J., Bratton D. L., Henson P. M. (1992). Exposure of phosphatidylserine on the surface of apoptotic lymphocytes triggers specific recognition and removal by macrophages.. J Immunol.

[PDF_00531] Falciani M., Gori A. M., Fedi S., Chiarugi L., Simonetti I., Dabizzi R. P., Prisco D., Pepe G., Abbate R., Gensini G. F. (1998). Elevated tissue factor and tissue factor pathway inhibitor circulating levels in ischaemic heart disease patients.. Thromb Haemost.

[PDF_00535] Fischer E. G., Ruf W., Mueller B. M. (1995). Tissue factor-initiated thrombin generation activates the signaling thrombin receptor on malignant melanoma cells.. Cancer Res.

[PDF_00538] Greeno E. W., Bach R. R., Moldow C. F. (1996). Apoptosis is associated with increased cell surface tissue factor procoagulant activity.. Lab Invest.

[PDF_00540] Hamada K., Kuratsu J., Saitoh Y., Takeshima H., Nishi T., Ushio Y. (1996). Expression of tissue factor correlates with grade of malignancy in human glioma.. Cancer.

[PDF_00543] Imamura T., Iyama K., Takeya M., Kambara T., Nakamura S. (1993). Role of macrophage tissue factor in the development of the delayed hypersensitivity reaction in monkey skin.. Cell Immunol.

[PDF_00546] Kakkar A. K., DeRuvo N., Chinswangwatanakul V., Tebbutt S., Williamson R. C. (1995). Extrinsic-pathway activation in cancer with high factor VIIa and tissue factor.. Lancet.

[PDF_00549] Kakkar A. K., Lemoine N. R., Scully M. F., Tebbutt S., Williamson R. C. (1995). Tissue factor expression correlates with histological grade in human pancreatic cancer.. Br J Surg.

[PDF_00552] Koomägi R., Volm M. (1998). Tissue-factor expression in human non-small-cell lung carcinoma measured by immunohistochemistry: correlation between tissue factor and angiogenesis.. Int J Cancer.

[PDF_00555] Koyama T., Nishida K., Ohdama S., Sawada M., Murakami N., Hirosawa S., Kuriyama R., Matsuzawa K., Hasegawa R., Aoki N. (1994). Determination of plasma tissue factor antigen and its clinical significance.. Br J Haematol.

[PDF_00559] Masuda M., Nakamura S., Murakami T., Komiyama Y., Takahashi H. (1996). Association of tissue factor with a gamma chain homodimer of the IgE receptor type I in cultured human monocytes.. Eur J Immunol.

[PDF_00562] Miller D. L., Yaron R., Yellin M. J. (1998). CD40L-CD40 interactions regulate endothelial cell surface tissue factor and thrombomodulin expression.. J Leukoc Biol.

[PDF_00565] Misumi K., Ogawa H., Yasue H., Soejima H., Suefuji H., Nishiyama K., Takazoe K., Kugiyama K., Tsuji I., Kumeda K. (1998). Comparison of plasma tissue factor levels in unstable and stable angina pectoris.. Am J Cardiol.

[PDF_00569] Morrissey J. H., Fakhrai H., Edgington T. S. (1987). Molecular cloning of the cDNA for tissue factor, the cellular receptor for the initiation of the coagulation protease cascade.. Cell.

[PDF_00572] Mueller B. M., Reisfeld R. A., Edgington T. S., Ruf W. (1992). Expression of tissue factor by melanoma cells promotes efficient hematogenous metastasis.. Proc Natl Acad Sci U S A.

[PDF_00575] Mueller B. M., Ruf W. (1998). Requirement for binding of catalytically active factor VIIa in tissue factor-dependent experimental metastasis.. J Clin Invest.

[PDF_00581] Nemerson Y., Bach R. (1982). Tissue factor revisited.. Prog Hemost Thromb.

[PDF_00583] Ollivier V., Bentolila S., Chabbat J., Hakim J., de Prost D. (1998). Tissue factor-dependent vascular endothelial growth factor production by human fibroblasts in response to activated factor VII.. Blood.

[PDF_00586] Ott I., Fischer E. G., Miyagi Y., Mueller B. M., Ruf W. (1998). A role for tissue factor in cell adhesion and migration mediated by interaction with actin-binding protein 280.. J Cell Biol.

[PDF_00589] Pendurthi U. R., Alok D., Rao L. V. (1997). Binding of factor VIIa to tissue factor induces alterations in gene expression in human fibroblast cells: up-regulation of poly(A) polymerase.. Proc Natl Acad Sci U S A.

[PDF_00592] Poulsen L. K., Jacobsen N., Sørensen B. B., Bergenhem N. C., Kelly J. D., Foster D. C., Thastrup O., Ezban M., Petersen L. C. (1998). Signal transduction via the mitogen-activated protein kinase pathway induced by binding of coagulation factor VIIa to tissue factor.. J Biol Chem.

[PDF_00596] Røttingen J. A., Enden T., Camerer E., Iversen J. G., Prydz H. (1995). Binding of human factor VIIa to tissue factor induces cytosolic Ca2+ signals in J82 cells, transfected COS-1 cells, Madin-Darby canine kidney cells and in human endothelial cells induced to synthesize tissue factor.. J Biol Chem.

[PDF_00601] Shigemori C., Wada H., Matsumoto K., Shiku H., Nakamura S., Suzuki H. (1998). Tissue factor expression and metastatic potential of colorectal cancer.. Thromb Haemost.

[PDF_00604] Shoji M., Hancock W. W., Abe K., Micko C., Casper K. A., Baine R. M., Wilcox J. N., Danave I., Dillehay D. L., Matthews E. (1998). Activation of coagulation and angiogenesis in cancer: immunohistochemical localization in situ of clotting proteins and vascular endothelial growth factor in human cancer.. Am J Pathol.

[PDF_00612] Slupsky J. R., Kalbas M., Willuweit A., Henn V., Kroczek R. A., Müller-Berghaus G. (1998). Activated platelets induce tissue factor expression on human umbilical vein endothelial cells by ligation of CD40.. Thromb Haemost.

[PDF_00615] Suefuji H., Ogawa H., Yasue H., Kaikita K., Soejima H., Motoyama T., Mizuno Y., Oshima S., Saito T., Tsuji I. (1997). Increased plasma tissue factor levels in acute myocardial infarction.. Am Heart J.

[PDF_00619] Takahashi H., Satoh N., Wada K., Takakuwa E., Seki Y., Shibata A. (1994). Tissue factor in plasma of patients with disseminated intravascular coagulation.. Am J Hematol.

[PDF_00622] Taniguchi T., Kakkar A. K., Tuddenham E. G., Williamson R. C., Lemoine N. R. (1998). Enhanced expression of urokinase receptor induced through the tissue factor-factor VIIa pathway in human pancreatic cancer.. Cancer Res.

[PDF_00626] Vrana J. A., Stang M. T., Grande J. P., Getz M. J. (1996). Expression of tissue factor in tumor stroma correlates with progression to invasive human breast cancer: paracrine regulation by carcinoma cell-derived members of the transforming growth factor beta family.. Cancer Res.

[PDF_00630] Wada H., Nakase T., Nakaya R., Minamikawa K., Wakita Y., Kaneko T., Ohiwa M., Deguchi K., Shirakawa S. (1994). Elevated plasma tissue factor antigen level in patients with disseminated intravascular coagulation.. Am J Hematol.

[PDF_00634] Wojtukiewicz M. Z., Zacharski L. R., Memoli V. A., Kisiel W., Kudryk B. J., Rousseau S. M., Stump D. C. (1989). Indirect activation of blood coagulation in colon cancer.. Thromb Haemost.

[PDF_00637] Yamamoto N., Watanabe T., Katsumata N., Omuro Y., Ando M., Fukuda H., Takue Y., Narabayashi M., Adachi I., Takashima S. (1998). Construction and validation of a practical prognostic index for patients with metastatic breast cancer.. J Clin Oncol.

[PDF_00641] Zacharski L. R., Schned A. R., Sorenson G. D. (1983). Occurrence of fibrin and tissue factor antigen in human small cell carcinoma of the lung.. Cancer Res.

[PDF_00644] Zhang Y., Deng Y., Luther T., Müller M., Ziegler R., Waldherr R., Stern D. M., Nawroth P. P. (1994). Tissue factor controls the balance of angiogenic and antiangiogenic properties of tumor cells in mice.. J Clin Invest.

[PDF_00648] Zhou L., Stordeur P., de Lavareille A., Thielemans K., Capel P., Goldman M., Pradier O. (1998). CD40 engagement on endothelial cells promotes tissue factor-dependent procoagulant activity.. Thromb Haemost.

